# Direct electrophilic *N*-trifluoromethylthiolation of amines with trifluoromethanesulfenamide

**DOI:** 10.3762/bjoc.9.270

**Published:** 2013-11-04

**Authors:** Sébastien Alazet, Kevin Ollivier, Thierry Billard

**Affiliations:** 1 Institute of Chemistry and Biochemistry (ICBMS – UMR CNRS 5246), Université de Lyon, Université Lyon 1, CNRS, 43 Bd du 11 novembre 1918 – 69622 Lyon, France; 2 CERMEP - in vivo imaging, Groupement Hospitalier Est, 59 Bd Pinel – 69003 Lyon, France

**Keywords:** amine, fluorine, organo-fluorine, trifluoromethanesulfenamide, trifluoromethylsulfanylamine, trifluoromethylthiolation

## Abstract

The CF_3_SN moiety is a substituent with interesting properties. However, there is no easy synthetic access to molecules bearing this group. The trifluoromethanesulfenamide is a new reagent for the electrophilic trifluoromethylthiolation which reacts easily with amines to obtain trifluoromethylsulfanylamines with good yields.

## Introduction

In past decades, fluorinated molecules have found more and more applications in a variety of fields, especially in the design of new compounds for medicinal chemistry or agrochemistry [1–9]. More recently, new substituents have emerged which associate the trifluoromethyl group with heteroatoms such as CF_3_O or CF_3_S. Because of its high hydrophobicity (Hansch parameter π_R_ = 1.44), the CF_3_S moiety is of particular interest [10]. Compounds with this group constitute important targets for applications in pharmaceuticals and agrochemicals [4,11–13].

The association of a CF_3_ group to more than one heteroatom is rarely described in literature. In particular, there are only a few investigations regarding the trifluoromethylsulfanylamine moiety (CF_3_SN). However, this group has found applications in agrochemical and medicinal chemistry [14–20]. From a physicochemical point of view, the CF_3_SN group possesses a Hansch’s hydrophobicity parameter π_R_ = 1.50 [21]. This value, slightly superior to the Hansch’s hydrophobicity parameter of the CF_3_S group, could be of great interest in the development of biological active compounds. Yet, the common synthetic route to these compounds use the highly toxic and gaseous CF_3_SCl [16,22–35].

## Results and Discussion

Several years ago, we have described an easy access to trifluoromethanesulfenamides [36], starting from DAST, Ruppert reagent, and primary amines [37]. However, even though this strategy gave good results with primary amines, secondary amines do not react under these conditions, thus limiting the access to a large panel of trifluoromethylsulfanylamines. The trifluoromethanesulfenamide **1a** is an efficient reagent for the electrophilic trifluoromethylthiolation of carbon nucleophiles [38–44]. Therefore, this reagent should react with amines to perform transamination reactions with secondary amines leading to various trifluoromethylsulfanylamines **3**. The reaction has been optimized with phenylpiperazine (**2a**) (Table 1).

**Table 1:** Reaction of phenylpiperazine (**2a**) with **1a** under basic conditions.

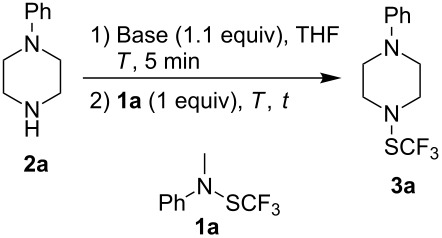				

Entry	Base	*T* (°C)	*t*	**3a** (%)^a^

1	BuLi	−78	15 min	65
2	BuLi	0	15 min	86
3	BuLi	0	3 h	84
4	NaH	0	15 min	0
5	Cs_2_CO_3_	80	2 h 30	0

^a^Crude yields determined by ^19^F NMR spectroscopy by using PhOCF_3_ as an internal standard.

**Figure 1:**
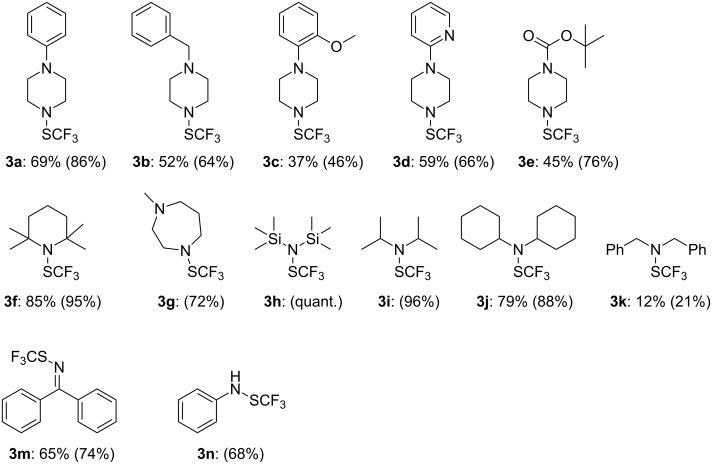
Transamination of **1a** with amines. (Isolated yields, in parentheses crude yields determined by ^19^F NMR with PhOCF_3_ as an internal standard).

After preliminary deprotonation of **2a** with BuLi, the trifluoromethanesulfenamide **1a** is added. The expected product **3a** is obtained in 15 min with good yield. To improve the kinetic of the reaction, the deprotonation and the transamination should be performed at 0 °C (Table 1, entries 1 and 2). Longer reaction times do not increase the yield, the reaction seems to be finished in 15 min (Table 1, entries 2 and 3). As previously observed in other works, the use of other bases with sodium or cesium cations is not efficient since only Li is a Lewis acid strong enough to activate **1a** [38,44]. These optimal conditions have been extended to selected amines **2** (Figure 1).

The reaction gives, in general, good yields with various secondary amines (**3a**–**k**). Because of their high volatility, some compounds (**3h** and **3i**) have not been isolated. Imines can be also trifluoromethylthiolated in good yields (**3m**). Even if our first developed method is compatible with primary amines [37], they can also react under these new conditions, as illustrated with the aniline (**3n**).

**Figure 2:**
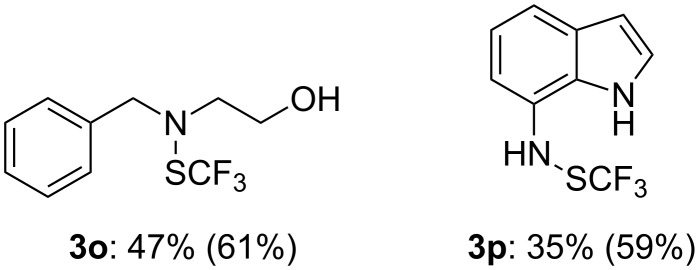
Reaction of **1a** with bis-nucleophiles. (Isolated yields, in parentheses crude yields determined by ^19^F NMR with PhOCF_3_ as an internal standard).

Amino alcohols and bis-amines can also be trifluoromethylthiolated, with the most nucleophile atom as a target (Figure 2). In this case, 2.1 equiv of BuLi are required and reaction times are loner (20 h).

**Figure 3:**
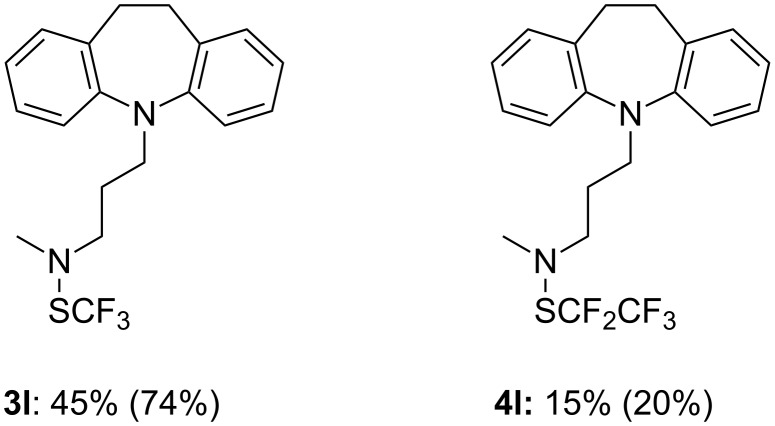
Synthesis of fluoroalkylthio analogs of imipramine. (Isolated yields, in parentheses crude yields determined by ^19^F NMR with PhOCF_3_ as an internal standard).

This new method was applied to synthesize a trifluoromethylthio analog (**3l**) of the well-known tricyclic antidepressant imipramine (Figure 3). Since the pentafluoroethyl analog **1b** of reagent **1a** has also been described previously, a pentafluoroethylthio analog of imipramine was synthesized (**4l**) (Figure 3). In the latter case, the obtained yield was lower, certainly due to the steric hindrance of the CF_3_CF_2_S moiety. The pharmacological properties of these new compounds are under investigation.

## Conclusion

In conclusion, the trifluoromethanesulfenamide **1a** is a very efficient reagent for the electrophilic trifluoromethylthiolation which can also react with amines to open a new access to trifluoromethylsulfanylamines. These compounds belong to a new class of products which may exhibit interesting properties for further applications – in particular in medicinal chemistry – owing to the characteristics of the CF_3_SN moiety.

## Supporting Information

File 1 Experimental procedure.
