# Supplementary material for: Direct electrophilic N-trifluoromethylthiolation of amines with trifluoromethanesulfenamide
Source: Beilstein J Org Chem. 2013 Nov 4;9:2354–7. doi: 10.3762/bjoc.9.270 (PMC3869241; doi:10.3762/bjoc.9.270)
Supplement: File 1 — Experimental procedure. [file Beilstein_J_Org_Chem-09-2354-s001.pdf]

Supporting Information

for

Direct electrophilic *N*-trifluoromethylthiolation of amines with trifluoromethanesulfenamide

Sébastien Alazet[1,2], Kevin Ollivier[1] and Thierry Billard*[1,2]

Address: [1]Institute of Chemistry and Biochemistry (ICBMS – UMR CNRS 5246), Université de Lyon, Université Lyon 1, CNRS, 43 Bd du 11 novembre 1918 – 69622 Lyon, France and [2]CERMEP - in vivo imaging, Groupement Hospitalier Est, 59 Bd Pinel – 69003 Lyon, France

Email: Thierry Billard - thierry.billard@univ-lyon1.fr

* Corresponding author

Experimental procedure

Table of Contents

General Information **S3**

Typical procedure **S3**

1-Phenyl-4-[(trifluoromethyl)sulfanyl]piperazine (**3a**) S3

1-Benzyl-4-[(trifluoromethyl)sulfanyl]piperazine (**3b**) S4

1-(2-Methoxyphenyl)-4-[(trifluoromethyl)sulfanyl]piperazine (**3c**) S4

1-(Pyridin-2-yl)-4-[(trifluoromethyl)sulfanyl]piperazine (**3d**) S4

tert-Butyl 4-[(trifluoromethyl)sulfanyl]piperazine-1-carboxylate (**3e**) S5

2,2,6,6-Tetramethyl-1-[(trifluoromethyl)sulfanyl]piperidine (**3f**) S5

1-Methyl-4-[(trifluoromethyl)sulfanyl]-1,4-diazepane (**3g**) S5

1,1,1-Trimethyl-*N*-[(trifluoromethyl)sulfanyl]-*N*-(trimethylsilyl)silanamine (**3h**) S5

N-Isopropyl-*N*-[(trifluoromethyl)sulfanyl]propan-2-amine (**3i**) S6

N-Cyclohexyl-*N*-[(trifluoromethyl)sulfanyl]cyclohexanamine (**3j**) S6

N-Benzyl-1-phenyl-*N*-[(trifluoromethyl)sulfanyl]methanamine (**3k**) S6

3-(10,11-Dihydro-5*H*-dibenzo[*b*,*f*]azepin-5-yl)-*N*-methyl-*N*-[(trifluoromethyl)sulfanyl]propan-1-amine (**3l**) . S6

3-(10,11-Dihydro-5*H*-dibenzo[*b*,*f*]azepin-5-yl)-*N*-methyl-*N*-[(pentafluoroethyl)sulfanyl]propan-1-amine (**4l**) S7

Diphenylmethanone *S*-(trifluoromethyl)thioxime (**3m**) S7

N-[(Trifluoromethyl)sulfanyl]aniline (**3n**) S7

2-{Benzyl[(trifluoromethyl)sulfanyl]amino}ethanol (**3o**) S7

N-[(Trifluoromethyl)sulfanyl]-1*H*-indol-7-amine (**3p**) S8

References **S8**

General Information

All reactions requiring anhydrous conditions were conducted in flame-dried glass apparatus under an atmosphere of nitrogen. THF was freshly distilled from sodium benzophenone ketyl prior to use. Commercial reagents were used as supplied.

Nuclear magnetic resonance spectra were recorded a Bruker AV 400 spectrometer at 400 MHz (^{1}H NMR), 100 MHz (^{13}C NMR), 389 MHZ (^{19}F NMR) in $CDCl_3$. All chemical shift values are reported in ppm (δ) relative to tetramethylsilane (TMS) for ^{1}H and ^{13}C NMR spectra and to $CFCl_3$ for ^{19}F NMR spectra and coupling constant (*J*) in Hertz.

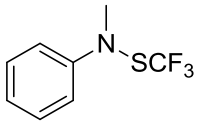

1a

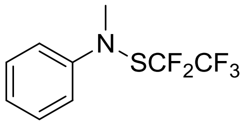

1b

Typical procedure

A dry and nitrogen-flushed-10 mL round bottom flask equipped with a magnetic stirrer and a septum was charged with amine **2** (0.5 mmol, 1 equiv) and was evacuated and refilled with nitrogen three times. Dry THF (0.5 mL) was added and the reaction flask was again evacuated and refilled with nitrogen three times. The reaction mixture was cooled to 0 °C, and BuLi solution (0.55 or 1.05 mmol, 2.45 M in THF, 1.1 or 2.1 equiv) was added. After 5 min of stirring, **1a** or **1b** (0.5 mmol, 1 equiv) was added at 0 °C and the reaction was checked by ^{19}F NMR with $PhOCF_3$ as internal standard. Reaction was quenched with saturated aqueous $NaHCO_3$ and EtOAc was added. The organic phase was washed with water, dried over Na_2SO_4, and concentrated in vacuo. The crude residue was purified by flash chromatography to give the expected product.

1-Phenyl-4-[(trifluoromethyl)sulfanyl]piperazine (3a)

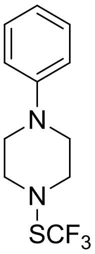

(Chromatographic solvent = 95/5 : Cyclohexane/EtOAc)
Translucent liquid.
^{1}H NMR: δ = 7.24 (m, 2H), 6.84 (m, 3H), 3.38 (m, 4H), 3.19 (m, 4H).
^{13}C NMR: δ = 151.3, 129.3, 130.6 (q, ^{1}J (C,F) = 321 Hz), 120.4, 116.8, 52.3, 50.8.
^{19}F NMR: δ = -46.53 (s, 3F).
Elemental analysis calcd (%) for $C_{11}H_{13}F_3N_2S$: C 50.37, H 5.00, N 10.68, S 12.22; Found: C 50.14, H 4.71, N 10.59, S 12.02.

1-Benzyl-4-[(trifluoromethyl)sulfanyl]piperazine (3b)

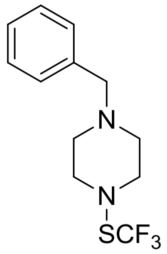

(Chromatographic solvent = Cyclohexane/EtOAc : 95/5)
Yellow liquid.
^{1}H NMR: δ =7.28-7.10 (m, 5H), 3.41 (s, 2H), 3.18 (m, 4H), 2.37 (m, 4H).
^{13}C NMR: δ = 137.8, 131.5 (q, ^{1}J (C,F) = 322 Hz), 129.2, 128.4, 127.3, 62.9, 57.1, 54.0.
^{19}F NMR: δ = -46.73 (s, 3F).
Elemental analysis calcd (%) for $C_{12}H_{15}F_3N_2S$: C 52.16, H 5.47, N 10.14, S 11.60; Found: C 52.45, H 5.37, N 9.97, S 11.45.

1-(2-Methoxyphenyl)-4-[(trifluoromethyl)sulfanyl]piperazine (3c)

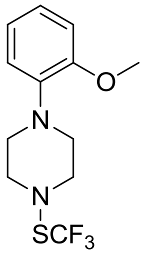

(Chromatographic solvent = Cyclohexane/EtOAc : 95/5)
Pale Yellow liquid.
^{1}H NMR: δ = 6.99-6.75 (m, 5H), 3.79 (s, 3H), 3.37 (m, 4H), 3.00 (m, 4H).
^{13}C NMR: δ = 152.3, 140.8, 131.5 (q, ^{1}J (C,F) = 323 Hz), 123.5, 121.1, 118.5, 111.3, 57.3, 55.5, 51.9.
^{19}F NMR: δ = -46.60 (s, 3F).
Elemental analysis calcd (%) for $C_{12}H_{15}F_3N_2OS$: C 49.30, H 5.17, N 9.58, S 10.97; Found: C 49.49, H 4.93, N 9.49, S 10.87.

1-(Pyridin-2-yl)-4-[(trifluoromethyl)sulfanyl]piperazine (3d)

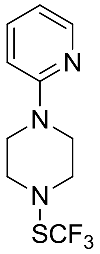

(Chromatographic solvent = Cyclohexane/EtOAc : 95/5)
Orange oil.
^{1}H NMR: δ = 8,18 (ddd, ^{3}J (H,H) = 4.9 Hz, ^{4}J (H,H) = 2.0 Hz, ^{5}J (H,H) = 0.9 Hz), 7.49 (ddd, ^{3}J (H-H) = 8.5 Hz, ^{3}J (H-H) = 6.9 Hz, ^{4}J (H,H) = 2.0 Hz,), 6.70-6.59 (m, 2H), 3.58 (s large, 4H), 3.36 (m, 4H).
^{13}C NMR: δ = 159.2, 148.1, 137.8, 131.4 (q, ^{1}J (C,F) = 320 Hz), 113.9, 107.4, 56.8, 46.5.
^{19}F NMR: δ = -46.63
Elemental Analysis calcd (%) for $C_{10}H_{12}F_3N_3S$: C, 45.62; H, 4.59; N, 15.96; S, 12.18; Found: C 45.87, H 4.29, N 16.1, S 12.1.

tert-Butyl 4-[(trifluoromethyl)sulfanyl]piperazine-1-carboxylate (3e)

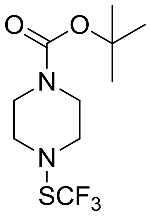

(Chromatographic solvent = Cyclohexane/EtOAc : 95/5)
Pale yellow liquid.
^{1}H NMR: δ = 3.46 (m, 4H), 3.21 (m, 4H), 1.48 (s, 9H).
^{13}C NMR: δ = 154.5, 131. (q, ^1J (C,F) = 325 Hz) ; 80.3, 56.9, 44(.7, 28.5.
^{19}F NMR: δ = -46.79 (s, 3F).
Elemental Analysis calcd (%) for $C_{10}H_{17}F_3N_2O_2S$: C, 41.95; H, 5.98; N, 9.78; S, 11.20; Found: C 42.27, H 6.26, N 9.87, S 11.49.

2,2,6,6-Tetramethyl-1-[(trifluoromethyl)sulfanyl]piperidine (3f)

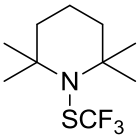

(Chromatographic solvent = Pentane)
Translucent liquid.
^{1}H NMR: δ = 1.67-1.48 (m, 6H), 1.28 (s, 6H), 1.26 (s, 6H).
^{13}C NMR: δ = 129.9 (q, ^1J (C,F) = 320 Hz), 60.12, 40.73, 25..65, 17.21.
^{19}F NMR: δ = -54.56 (s, 3F).
Elemental analysis calcd (%) for $C_{10}H_{18}F_3NS$: C 49.77, H 7.52, N 5.80, S 13.29; Found: C 50.06, H 7.80, N 5.57, S 12.98.

1-Methyl-4-[(trifluoromethyl)sulfanyl]-1,4-diazepane (3g)

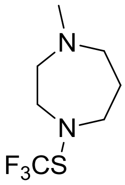

^{1}H NMR: δ = 3.45 (m, 4H), 2.58 (m, 4H), 2.37 (s, 3H), 1.83 (m, 2H).
^{19}F NMR: δ = -51.59 (s, 3F).

1,1,1-Trimethyl-*N*-[(trifluoromethyl)sulfanyl]-*N*-(trimethylsilyl)silanamine (3h)

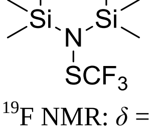

^{19}F NMR: δ = -52.37 (s, 3F) (in accordance with literature [1])

N-Isopropyl-N-[(trifluoromethyl)sulfanyl]propan-2-amine (3i)

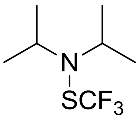

^{19}F NMR: δ = -53.42 (s, 3F) (in accordance with literature [1]).

N-Cyclohexyl-N-[(trifluoromethyl)sulfanyl]cyclohexanamine (3j)

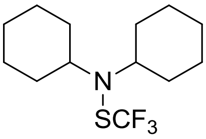

(Chromatographic solvent = Pentane)
Translucent oil.
^{1}H NMR: δ = 2.88 (m, 2H), 1.81-1.63 (m, 8H), 1.61-0.79 (m, 12H).
^{13}C NMR: δ = 129.9 (q, ^{1}J (C,F) = 332 Hz), 64.94, 33.3, 33.0, 26.3, 26.2, 25.65.
^{19}F NMR: δ = -53.81 (s, 3F).
Elemental analysis calcd (%) for $C_{13}H_{22}F_3NS$: C 55.49, H 7.88, N 4.98, S 11.40; Found: C 55.65, H 7.75, N 4.66, S 11.34.
(already described in literature [2]).

N-Benzyl-1-phenyl-N-[(trifluoromethyl)sulfanyl]methanamine (3k)

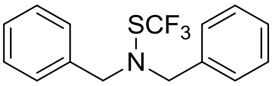

(Chromatographic solvent = Cyclohexane/EtOAc : 99/1)
Translucent oil.
^{1}H NMR: δ = 7.49-7.37 (m, 10H), 4.35 (s, 4H). (in accordance with literature [3]).
^{13}C NMR: δ = 137.9, 131.9(q, ^{1}J (C, F) = 323 Hz), 129.4, 129.0, 128.3, 62.4.
^{19}F NMR: δ = -47.72 (s, 3F).
Elemental analysis calcd (%) for $C_{15}H_{14}F_3NS$: C 60.59, H 4.75, N 4.71, S 10.78; Found: C 60.35, H 5.12, N 5.00, S 10.50.

3-(10,11-Dihydro-5H-dibenzo[b,f]azepin-5-yl)-N-methyl-N-[(trifluoromethyl)sulfanyl]propan-1-amine (3l)

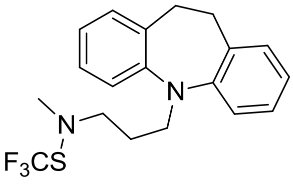

(Chromatographic solvent = Cyclohexane/EtOAc : 98/2)
Yellow oil.
^{1}H NMR: δ = 7.21-6.99 (m, 6H), 7.01-6.83 (m, 2H), 3.75 (t, ^{3}J (H,H) = 6.7 Hz, 2H), 3.17 (s, 4H), 3.09 (t, ^{3}J (H,H) = 6.7 Hz, 2H), 2.88 (s, 3H), 1.84 (m, 2H).
^{13}C NMR: δ = 148.3, 134.4, 131.7 (q, ^{1}J (C, F) = 320 Hz), 130.0, 126.5, 122.7, 119.9, 58.2, 47.7, 47.4, 32.3, 26.5.
^{19}F NMR: δ = -47.52 (s, 3F).
Elemental analysis calcd (%) for $C_{19}H_{21}F_3N_2S$: C 62.28, H 5.78, N 7.64, S 8.75; Found: C 62.57, H 5.98, N 7.46, S 8.57.

3-(10,11-Dihydro-5*H*-dibenzo[*b*,*f*]azepin-5-yl)-*N*-methyl-*N*-[(pentafluoroethyl)sulfanyl]propan-1-amine (4l)

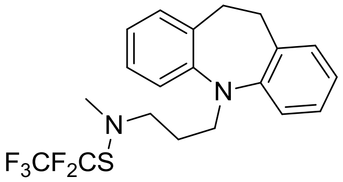

(Chromatographic solvent = Pentane/ EtOAc : 99/1)
Yellow oil.
^{1}H NMR: δ = 7.18-6.92 (m, 8H), 3.76 (t, ^{3}J (H,H) = 6.7 Hz, 2H), 3.18 (s, 4H), 3.14 (t, ^{3}J (H,H) = 6.7 Hz, 2H), 2.89 (s, 3H),1.85 (m, 2H).
^{13}C NMR: δ = 148.3, 134.4, 130.0, 126.5, 122,9 (tq, ^{1}J (C-F) = 287, ^{2}J (C-F) = 41, 2F), 122.7, 119.9, 118.7 (qt, ^{1}J (C-F) = 286, ^{2}J (C-F) = 37, 3F), 58.8, 47.6, 47.5, 32.3, 26.5.
^{19}F NMR: δ = -83.89 (t, ^{3}J (F-F) = 3.7Hz, 3F), -99.73 (t, ^{3}J (F-F) = 3.7, 2F).
Elemental analysis calcd (%) for $C_{20}H_{21}F_5N_2S$: C 57.68, H 5.08, N 6.73, S 7.70; Found: C 57.84, H 4.80, N 7.06, S 7.95.

Diphenylmethanone *S*-(trifluoromethyl)thioxime (3m)

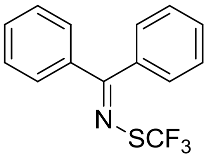

(Chromatographic solvent = Cyclohexane/EtOAc : 95/5)
Translucent liquid.
^{1}H NMR: δ = 7.61 (m, 2H), 7.54 (m, 3H), 7.45 (m, 1H), 7.37 (m, 2H), 7.31 (m, 2H).
^{13}C NMR: δ = 167.9, 137.8, 136.7, 131.3, 130.8, 129.6 (q, ^{1}J (C,F) = 311 Hz), 129.3, 128.4, 128.3, 127.1.
^{19}F NMR: δ = -50.70 (s, 3F).
Elemental analysis calcd (%) for $C_{14}H_{10}F_3NS$: C 59.78, H 3.58, N 4.98, S 11.40; Found: C 59.83, H 3.89, N 4.65, S 11.59.

***N*-[(Trifluoromethyl)sulfanyl]aniline (3n)**

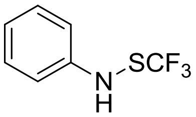

^{19}F NMR: δ = -52.96 (s, 3F) (in accordance with literature [4])

2-{Benzyl[(trifluoromethyl)sulfanyl]amino}ethanol (3o)

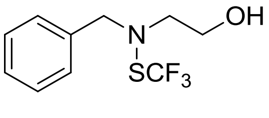

(Chromatographic solvent = Cyclohexane/EtOAc: 80/20)
Yellow liquid.
^{1}H NMR: δ = 7.39-7.30 (m, 5H), 4.35 (s, 2H), 3.75 (m, 2H), 3 .21 (m, 2H), 1.79 (s, 1H, OH).

^{13}C NMR: δ = 137.5, 132.8 (q, ^{1}J (C,F) = 311 Hz), 128.8, 128.6, 128.1, 64.7, 60.1, 58.9.
^{19}F NMR: δ =-50.71 (s, 3F).
Elemental analysis calcd (%) for $C_{10}H_{12}F_3NOS$: C 47.80, H 4.81, N 5.57, S 12.76; Found: C 47.98, H 4.54, N 5.85, S 12.57.

***N*-[(Trifluoromethyl)sulfanyl]-1*H*-indol-7-amine (3p)**

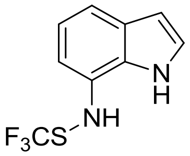

(Chromatographic solvent = Cyclohexane/EtOAc: 80/20)
Orange oil.
^{1}H NMR: δ = 8.23 (s, 1H, H-N), 7.23- 7.14 (m, 2H), 7.08 (d, ^{3}J (H-H) = 8.6 Hz, 1H), 7.06 (d, ^{3}J (H-H) = 8.6 Hz, 1H), 6.51 (m, 1H), 5.37 (s, 1H, H-N).
^{13}C NMR: δ= 137.9, 136.8, 129,6 (q, ^{1}J (C,F) = 323 Hz), 123.5, 123.1, 118.1, 105.3, 103.9, 98.2.
^{19}F NMR: δ = -53,19 (s, 3F).
Elemental analysis calcd (%) for $C_9H_7F_3N_2S$: C 46.55, H, 3.04, N 12.06, S 13.81; Found: C 46.84, H 2.72, N 12.39, S 13.96.

References

[1] Kolasa, A.; Lieb, M. *J. Fluor. Chem*. **1995**, 70, 45-47.

[2] Yagupol'skii, L. M.; Bezdudnyi, A. V.; Yagupol'skii, Y. L. *Russ. J. Org. Chem.* **2006,** *42*, 1275-1279. The NMR descriptions given in this reference are different of our experimental data. However, all our analyses seem also in accordance with the product structure.

[3] Bacque, E.; El-Ahmad, Y.; Billard, T.; Langlois, B.; Ferry, A. Process of preparation of fluorinated sulfanylamides and sulfinamidines from aminosulfur trifluorides and silanes, and their use as perfluoroalkylsulfanylation agent and biological active agents. WO2008110698A2, 2008.

[4] Ferry, A.; Billard, T.; Langlois, B. R.; Bacque, E. *J. Org. Chem.* **2008,** *73*, 9362-9365.
